# Nanoantenna structures for the detection of phonons in nanocrystals

**DOI:** 10.3762/bjnano.9.246

**Published:** 2018-10-05

**Authors:** Alexander G Milekhin, Sergei A Kuznetsov, Ilya A Milekhin, Larisa L Sveshnikova, Tatyana A Duda, Ekaterina E Rodyakina, Alexander V Latyshev, Volodymyr M Dzhagan, Dietrich R T Zahn

**Affiliations:** 1 Rzhanov Institute of Semiconductor Physics RAS, Lavrentiev Ave. 13, 630090 Novosibirsk, Russia; 2 Novosibirsk State University, Pirogov 2, 630090 Novosibirsk, Russia; 3 Rzhanov Institute of Semiconductor Physics RAS, Novosibirsk Branch “TDIAM”, Lavrentiev Ave. 2/1, Novosibirsk 630090, Russia; 4 V. E. Lashkaryov Institute of Semiconductor Physics of National Academy of Sciences of Ukraine, Prospekt Nauky 41, 03028 Kyiv, Ukrain; 5 Semiconductor Physics, Technische Universitaet Chemnitz, 09126, Chemnitz, Germany

**Keywords:** localized surface plasmon resonance, metal nanoclusters, nanoantenna, phonons, semiconductor nanocrystals, surface-enhanced infrared absorption

## Abstract

We report a study of the infrared response by localized surface plasmon resonance (LSPR) modes in gold micro- and nanoantenna arrays with various morphologies and surface-enhanced infrared absorption (SEIRA) by optical phonons of semiconductor nanocrystals (NCs) deposited on the arrays. The arrays of nano- and microantennas fabricated with nano- and photolithography reveal infrared-active LSPR modes of energy ranging from the mid to far-infrared that allow the IR response from very low concentrations of organic and inorganic materials deposited onto the arrays to be analyzed. The Langmuir–Blodgett technology was used for homogeneous deposition of CdSe, CdS, and PbS NC monolayers on the antenna arrays. The structural parameters of the arrays were confirmed by scanning electron microscopy. 3D full-wave electromagnetic simulations of the electromagnetic field distribution around the micro- and nanoantennas were employed to realize the maximal SEIRA enhancement for structural parameters of the arrays whereby the LSPR and the NC optical phonon energies coincide. The SEIRA experiments quantitatively confirmed the computational results. The maximum SEIRA enhancement was observed for linear nanoantennas with optimized structural parameters determined from the electromagnetic simulations. The frequency position of the feature’s absorption seen in the SEIRA response evidences that the NC surface and transverse optical phonons are activated in the infrared spectra.

## Introduction

Surface-enhanced infrared absorption (SEIRA) by organic species placed on metal surfaces proposed in [1] has been the subject of intensive research over the past years [2–4]. Several groups showed that the IR response by organic molecules can be significantly enhanced in a variety of organic systems by depositing the molecules on nanostructured surfaces of noble metals (Ag, Au, Cu, etc*.*) [5–7]. The origin of the IR signal enhancement is the localized electromagnetic field of plasmons excited near metallic surfaces. In the case of flat metal films, the IR response from an organic molecule in the plasmon field can be increased by a factor of 10^3^ [8]. For island metal films, the enhancement was found to depend on the island aspect ratio and the size of the gap between nanoclusters [9–10]. It is worth noting that SEIRA is maximized for elongated metal nanoclusters (nanorods or nanoantennas) with a high aspect ratio (length-to-width ratio) and a small gap between nanoclusters [11]. However, stochastical distribution of metal nanoclusters in terms of size, shape, and orientation reduces the IR enhancement and impedes the study of SEIRA regularities. Further progress was achieved with the development of nanolithography techniques which made possible the fabrication of arrays of metal nanoantennas with structural parameters that were well-controlled at the nanometer scale [12–13]. The most common, linear-shaped nanoantennas exhibit two LSPR modes polarized parallel and perpendicular to the nanoantenna axis (herein, referred to as the longitudinal and transverse modes, respectively). The longitudinal mode has a LSPR energy in the IR spectral range and is utilized for SEIRA experiments. This energy can be gradually tuned from near- to far-infrared (or terahertz) via increasing the nanoantenna length [14–15]. The transverse mode has a much shorter wavelength – appearing in the visible spectral range and of interest for optical spectroscopy [16].

It was shown that regular linear nanoantennas fabricated by nanolithography demonstrate enhancement of the SEIRA signal from vibrational modes in organic molecules such as octadecanethiol (ODT) [17] and 4,4'-bis(*N*-carbazolyl)-1,1'-biphenyl (CBP) by a factor of 10^5^ [18]. The highest SEIRA response was obtained by adjusting the LSPR energy of the nanoantennas to the energy of the vibrational modes [17]. The high sensitivity of SEIRA to vibrational modes allowed the detection of organic and biological substances of extremely low concentrations. For example, Adato et al. demonstrated the detection of a concentration as low as 145 protein molecules per nanoantenna [19]. SEIRA has been extended to the far-IR spectral range for which special nanoscale slot-antenna arrays were designed to determine glucose and fructose concentrations in solutions, including market beverages [20] with concentrations as low as 10 mg/dL.

Despite the significant progress in SEIRA of organic molecules at ultra-low concentrations, the plasmonic enhancement of IR absorption by inorganic nanomaterials like nanocrystals (NCs) has not been extensively examined to date. Recently, A. Toma et al. [21] published the first report on SEIRA for detection of an optical phonon mode (so-called Froehlich mode) from a monolayer of CdSe NCs deposited on Au nanoantenna arrays. The SEIRA enhancement induced by the nanoantennas was estimated to be as high as 1 × 10^6^. In our earlier papers we demonstrated SEIRA in the mid- and far-infrared for both organic molecules and inorganic NCs deposited on Au linear nanoantenna arrays fabricated on Si substrates by nanolithography [22–23]. The influence of a thin SiO_2_ layer (0–100 nm) beneath the nanoantenna arrays on the LSPR energy, as well as the LSP penetration depth into SiO_2_ were established [12]. We also showed that diffraction modes in linear Au nanoantenna arrays propagating along the Si surface and perpendicular to the nanoantennas [24] can be effectively employed for further enhancement of SEIRA by optical phonons in semiconductor NCs [23]. The electromagnetic ﬁeld distribution around the linear antennas was calculated using three-dimensional electrodynamics simulations, where the maximal SEIRA enhancement was realized for an array period of about 15 μm when the energy of a diffraction mode coincides with that of the LSPR mode [23].

Besides linear nanoantennas, plasmonic structures with more sophisticated geometries have been probed in SEIRA experiments. These structures include fan-shaped nanoantennas [25], H- and U-shaped nanoantennas [26], Jerusalem-cross-shaped nanoapertures [27], nanorings [28], and nanoslits [29]. A detailed description of various nanoantenna geometries can be found in the comprehensive review [30].

In this paper, we report on a systematic study of the effect of SEIRA by the phonon response from monolayers of CdSe, CdS, and PbS NCs deposited on periodic arrays of Au nano- and microantennas of linear and H-like shapes having the LSPR energy close to the surface optical phonon energy of in the corresponding NCs. It should be mentioned that although the same conceptual framework and methodological toolkit were used in this paper as in [23] (including sample design, and analytical and computational analysis approaches), we probe and compare the plasmonic properties of nanoantennas with various sample geometry as well as their SEIRA performance. We show that even though SEIRA is evidenced for all plasmonic micro- and nanostructures under investigation, the maximal local enhancement of the NC phonon response is obtained for NCs deposited on the linear nanoantennas with structural parameters optimized via 3D full-wave electromagnetic simulations.

## Experimental

The uniform, periodic, linear, Au nanoantenna arrays with the overall dimensions of 3 × 3 mm^2^, differing in nanoantenna length and lateral periodicity, were fabricated on bare Si(001) substrates and substrates covered with SiO_2_ layers of different (5–100 nm) thicknesses by direct electron beam writing (Raith-150, Germany) as described earlier [23,31]. For H-shaped nanoantennas, additional Au symmetric cross-arms were introduced on the nanoantenna edges of the linear nanoantennas.

The microantenna arrays with the overall dimensions of 8 × 8 mm^2^ were patterned on Si(001) substrates using a conventional photolithography technique. The width (height) of microantennas was chosen to be 4 µm (50 nm), while the antenna length varied in the range of 7–31 µm. The optimal values of the latter were determined numerically using 3D full-wave simulations in the ANSYS Electromagnetics Suite R18 software [32] to adjust the plasmon resonance to a specific wavelength in the far-infrared (terahertz) spectral region.

The monolayers (MLs) of CdS and PbS NCs fabricated and MLs of commercially available colloidal CdSe NCs were homogeneously deposited on the prepared plasmonic substrates using the Langmuir–Blodgett (LB) technique as described earlier [23,33–35].

The size, shape, and areal density of NCs, as well as the structural parameters of nanoantennas (length, width, and lateral periodicity) were measured by scanning electron microscopy (SEM) using the same Raith-150 system at 10 kV acceleration voltage, 30 µm aperture, and 6 mm working distance. The structural parameters of the microantennas were controlled by optical microscopy. The diameter of the CdS and PbS NCs determined from SEM measurements was found to be 4.5 ± 1.5 nm and 7 ± 3 nm, respectively [23], while the diameter of the colloidal CdSe NCs purchased from Lumidot was 5.0 ± 0.3 nm.

FTIR transmission measurements of Au nano- and microantenna arrays were carried out in the spectral range of 30–5000 cm^−1^ using a Bruker Vertex 80v Fourier transform spectrometer. The IR spectra were recorded for different angles of incidence (from 0 to 55°) and polarizations (TE and TM). For further evaluation, the ratio of the IR transmission spectra corresponding to the light polarization along the nanoantenna axis and perpendicular to it was calculated and analyzed.

## Results and Discussion

### LSPR modes in nanoantennas with different morphology

**Figure 1:**
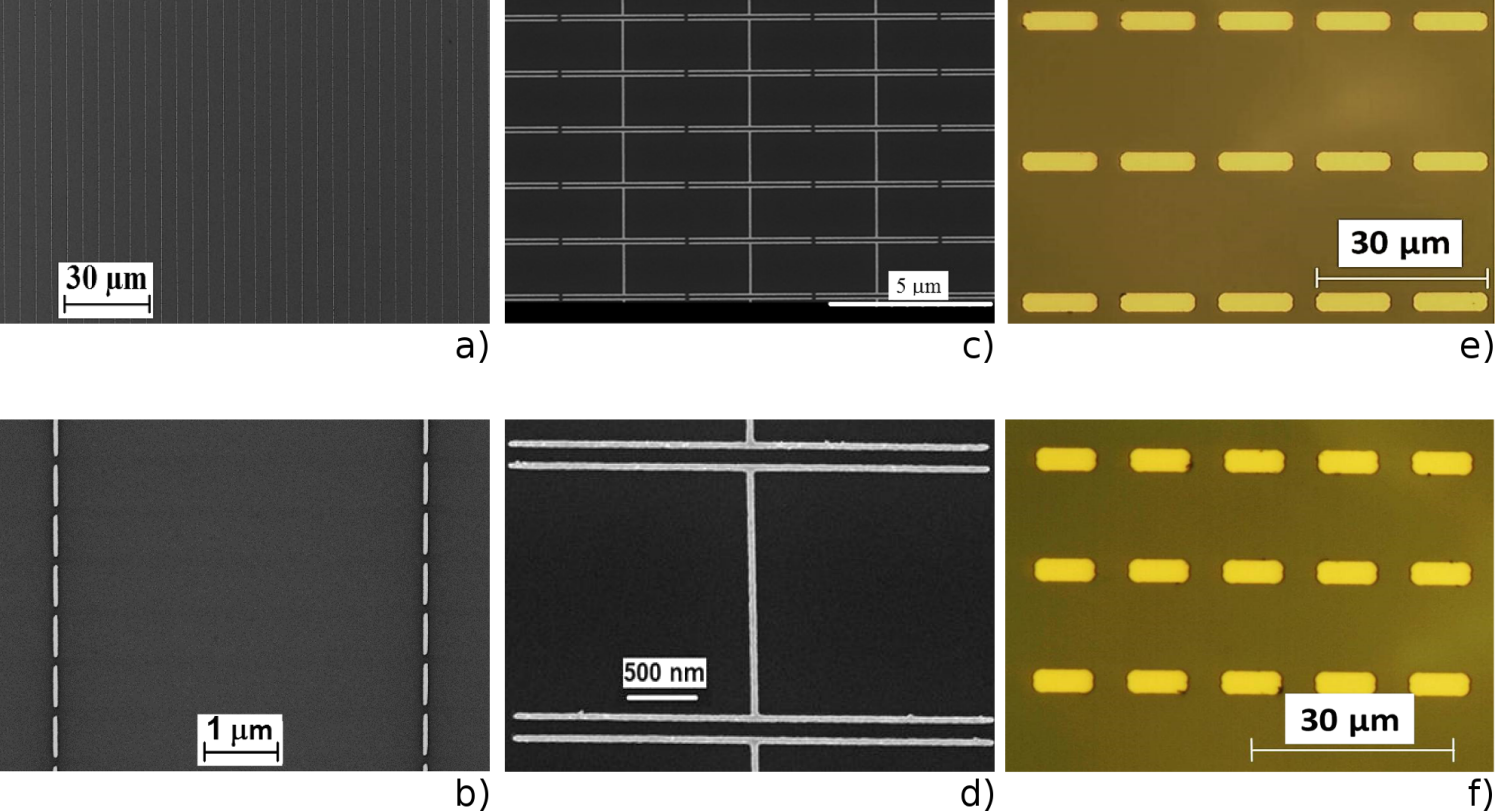
Representative SEM images of a) and b) linear and c) and d) H-shaped nanoantenna arrays with different resolution. e) and f) Optical images of the bare microantenna arrays of different lengths.

The typical SEM and optical images of nano- and microantenna arrays used as substrates for the deposition of NCs are shown in Figure 1. The length and transverse period of the nanoantennas in different arrays were changed, while the nanoantenna width and the inter-antenna gap were fixed to be about 100 nm.

**Figure 2:**
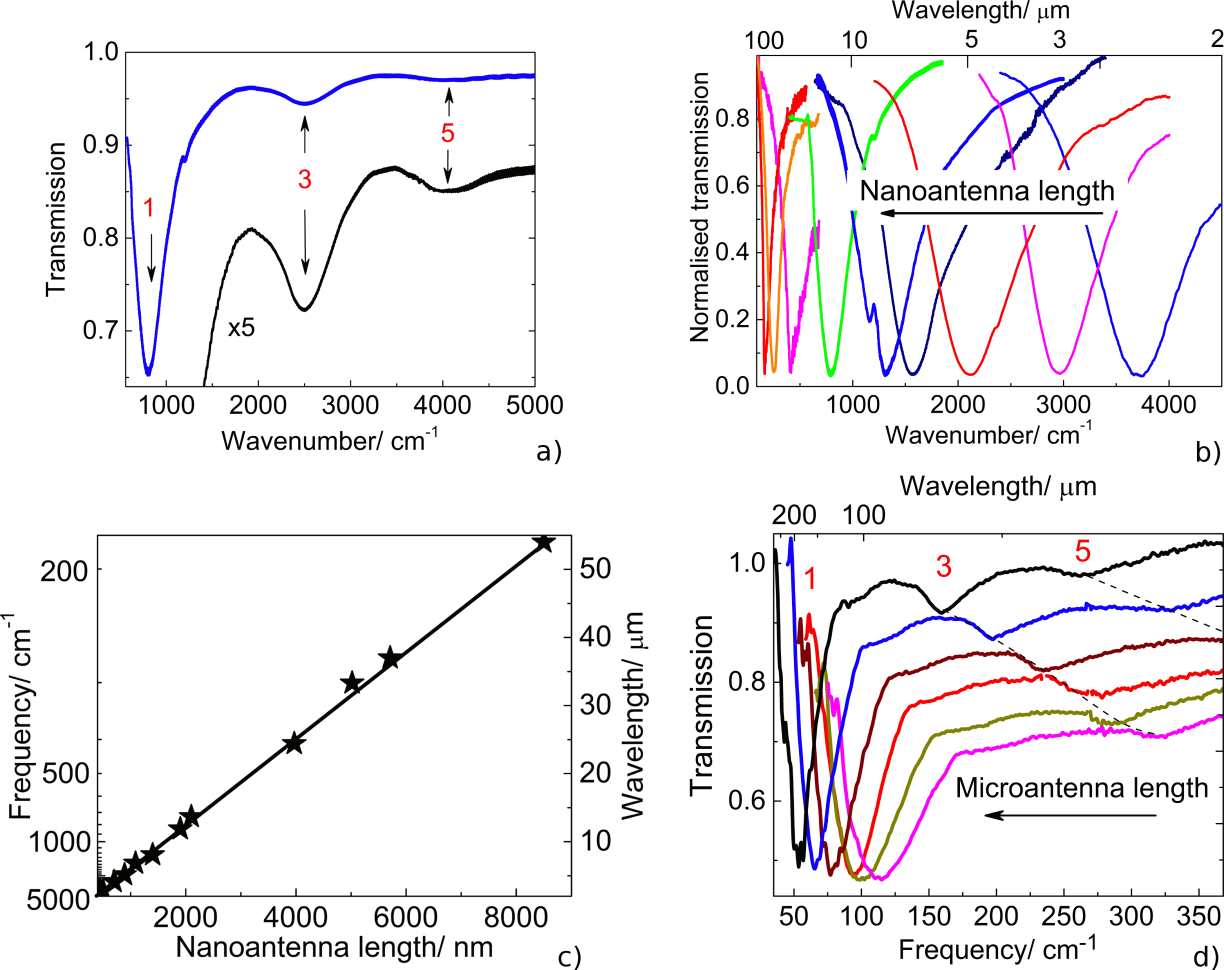
a) Typical IR transmission spectrum for the array of nanoantennas with a length of 1800 nm. The lower curve (black, labeled as ×5) shows the magnified IR spectrum (blue) to emphasize the higher-order plasmon modes. b) Normalized IR transmission spectra of linear antennas with different lengths (adopted from [12]). c) LSPR energy in nanoantenna arrays fabricated on bare Si surfaces as a function of the nanoantenna length (adopted from [12]). d) Normalized IR transmission spectra of the fabricated microantennas with different lengths (14.7; 16.4; 17.8; 20.2; 24.3; 30.6 µm). The numbers in the figures indicate the order of the LSPR modes.

In Figure 2a and 2b, the IR transmission spectra of the linear nanoantenna arrays fabricated with different nanoantenna lengths are presented. The spectra demonstrate distinct deep minima, the position of which corresponds to the LSPR energy. The weaker minima, corresponding to higher-order LSPR modes, are also observed at odd multiples (3rd and 5th) of the fundamental LSPR wavenumber (Figure 2a), whereas the even order LSPR modes remain inactive in the IR spectra recorded under normal incidence.

As it can be clearly seen from Figure 2b, the experimentally determined LSPR wavelength reveals a linear dependence on the antenna length in the investigated spectral range. The linear nanoantennas effectively couple to the incident electromagnetic waves once their wavelength coincides with the doubled antenna length [36]. This coupling also depends on the dielectric function of the surrounding medium that causes an increase in the LSPR energy when a thin SiO_2_ layer (with a thickness of 0.5–100 nm) is introduced beneath the nanoantennas [12].

Similar to nanoantenna arrays, the LSPR wavelength of the Au microantennas also undergoes a red shift with increasing the antenna length. However, this shift exhibits a nonlinear behavior owing to the fact that for short microantennas their length becomes comparable to the antenna width. Due to the nonlinear scaling, prior to fabricating the microantennas with the desired LSPR energy, additional 3D full-wave simulations were carried out.

The H-shaped nanoantennas possess two LSPR modes polarized parallel and perpendicular to the nanoantenna axis with frequencies located in the IR spectral region. It is worth noting that for the LSPR mode polarized parallel to the H-shaped nanoantennas, the enhancement of the electromagnetic field averaged over the total surface of the nanoantenna arrays can exceed that for linear nanoantenna arrays.

**Figure 3:**
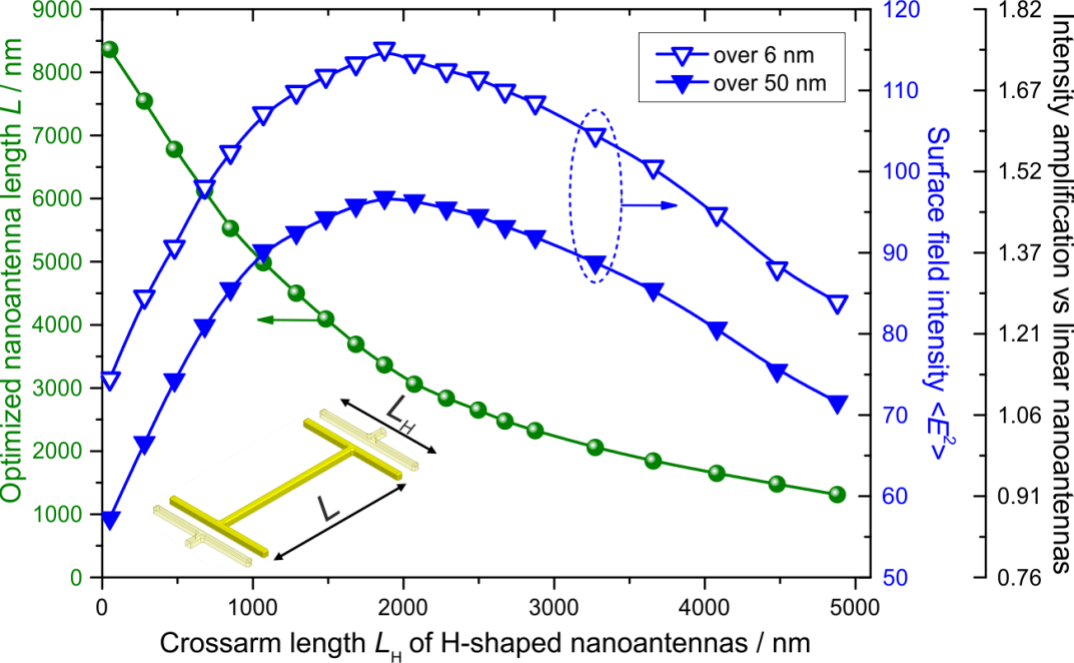
Optimized nanoantenna length *L* vs the transverse crossarm length *L*_H_ for H-shaped nanoantennas with the LSPR energy fixed at 190 cm^−1^ (circles) and the dependence of the averaged electric field intensity <*E*^2^> on *L*_H_ (triangles). The transverse spacing between antennas is chosen to coincide with the wavelength of the first 1st diffraction harmonics: *G_y_* = 15380 nm. The rightmost vertical scale represents <*E*^2^> normalized to that of linear nanoantennas optimized for 190 cm^−1^.

**Figure 4:**
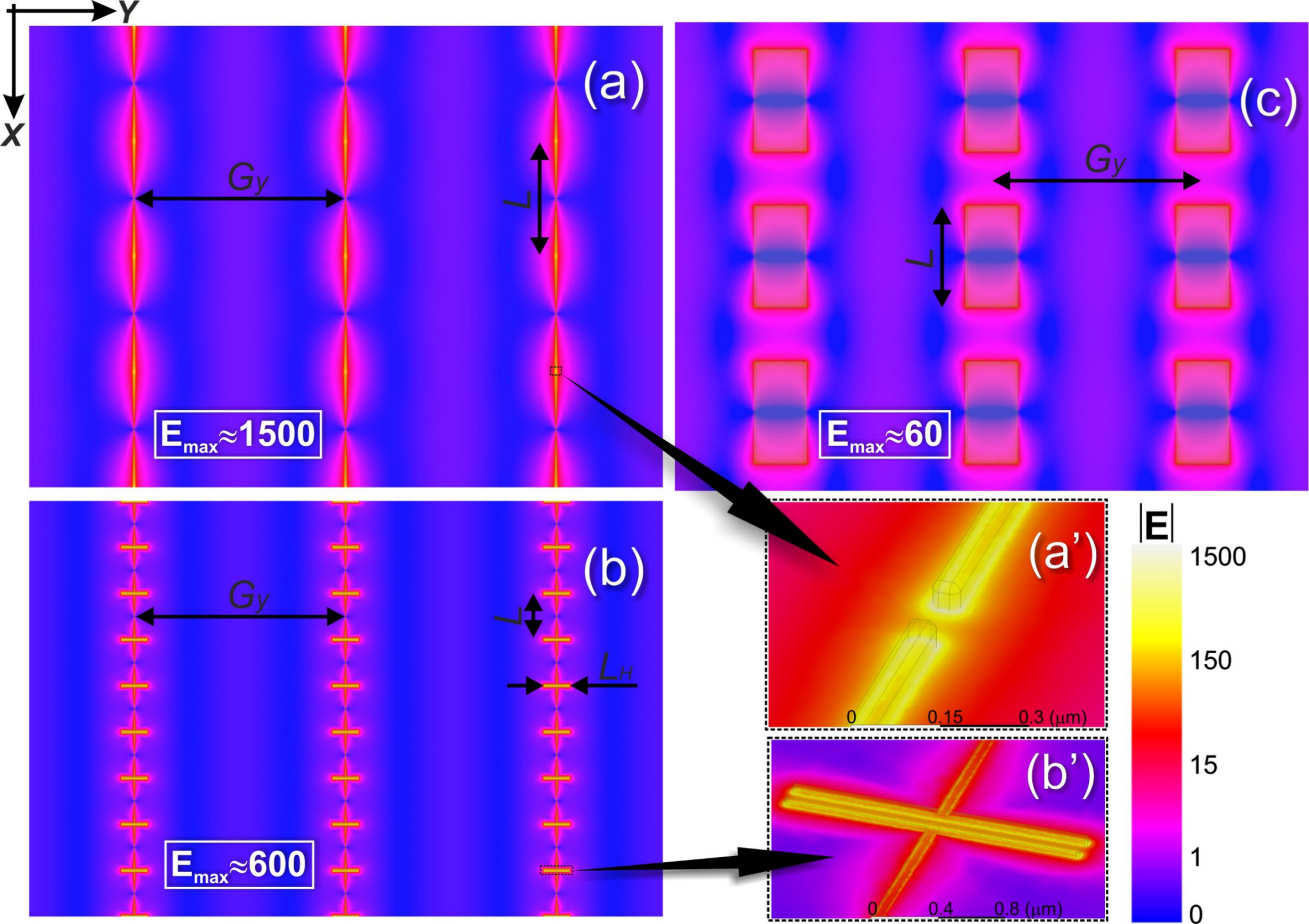
Distribution of the normalized LSPR electric field magnitude on top of the Si surface underlying the antenna arrays optimized for the LSPR energy of 190 cm^−1^: (a), (a’) linear nanoantennas, *L* = 8360 nm; (b), (b’) H-shaped nanoantennas, *L* = 3290 nm, *L*_H_ = 1880 nm; (c) linear microantennas, *L* = 7.7 µm. The transverse period *G_y_* = 15380 nm coinciding with the 1st diffraction lobe onset point is chosen for all the cases. The E-field is normalized to that when the antenna array is removed from Si surface, thereby displaying relative antenna-induced field amplification. The framed numbers indicate the peak values. Normal excitation, **E_0_** II **X**.

In this work, we propose the idea of using H-shaped nanoantennas instead of the linear-shaped ones to further enhance the averaged E-field intensity <*E*^2^> in the vicinity of nanoantennas. The H-shape is obtained by introducing symmetric cross-arms on the antenna edges as shown in Figure 3, thereby enabling control of the LSPR frequency through two length parameters: *L* and *L*_H_. Note that the cross-arms increase the intra-antenna capacitive coupling as compared to the linear nanoantennas. This results in smaller values of the length *L* versus the cross-arm-free case when fixing the LSPR frequency at a prescribed value. Despite the fact that, with all other parameters being equal, the linear antennas yield the highest peak magnitude of the local field among all geometries (see Figure 4 below), the smaller longitudinal unit cell size and quasi-uniform field distribution between the cross-arms of adjacent H-shaped antennas results in an increase of the <*E*^2^> value volumetrically averaged over the antenna height within the array unit cell. When increasing *L*_H_, such augmentation is to be manifested up to some limit below which the electric field becomes too small and incapable of compensating the unit cell size decrease. This effect is illustrated in Figure 3 where the optimized nanoantenna length *L* and the averaged electric field intensity <*E*^2^> are plotted as a function of the cross-arm length *L*_H_ for the example of H-shaped nanoantennas with the LSPR energy fixed at 190 cm^−1^. Optimization was carried out in the ANSYS EM Suite software; details of the electric field averaging procedure are described in [23]. When choosing the transverse spacing between nanoantennas *G_y_* we exploited the condition of superposing the LSPR wavelength λ_LSPR_ and the 1st diffraction harmonics excited in a Si wafer to maximize the E-field enhancement [23]: λ_LSPR_/*n*_Si_ = *G_y_* = 15380 nm, where *n*_Si_ = 3.421 is the refractive index of silicon. The results presented in Figure 3 prove: a) the existence of the optimal cross-arm length *L*_H_ (1880 nm) and b) higher <*E*^2^> values attainable for the H-shaped nanoantennas as compared to the linear ones. In the current example, the H-shaped antennas exhibit a maximal gain of 175% relative to that of the linear antennas.

Figure 4 illustrates the computed surface E-field distribution for three different types of antenna arrays optimized for the LSPR frequency of 190 cm^−1^ and fabricated in this work to compare their efficiency in terms of the field enhancement: linear nanoantennas (a, a’), H-shaped nanoantennas (b, b’), and linear microantennas (c). In all three cases the transverse period *G_y_* = 15380 nm was chosen to coincide with the 1st diffraction lobe onset point as explained above. The relative E-field peak enhancement reaches 1500 for the linear nanonantennas, while decreasing down to 600 and 60 for the H-shaped nanoantennas and the linear microantennas, respectively.

### Higher-order LSPR modes

**Figure 5:**
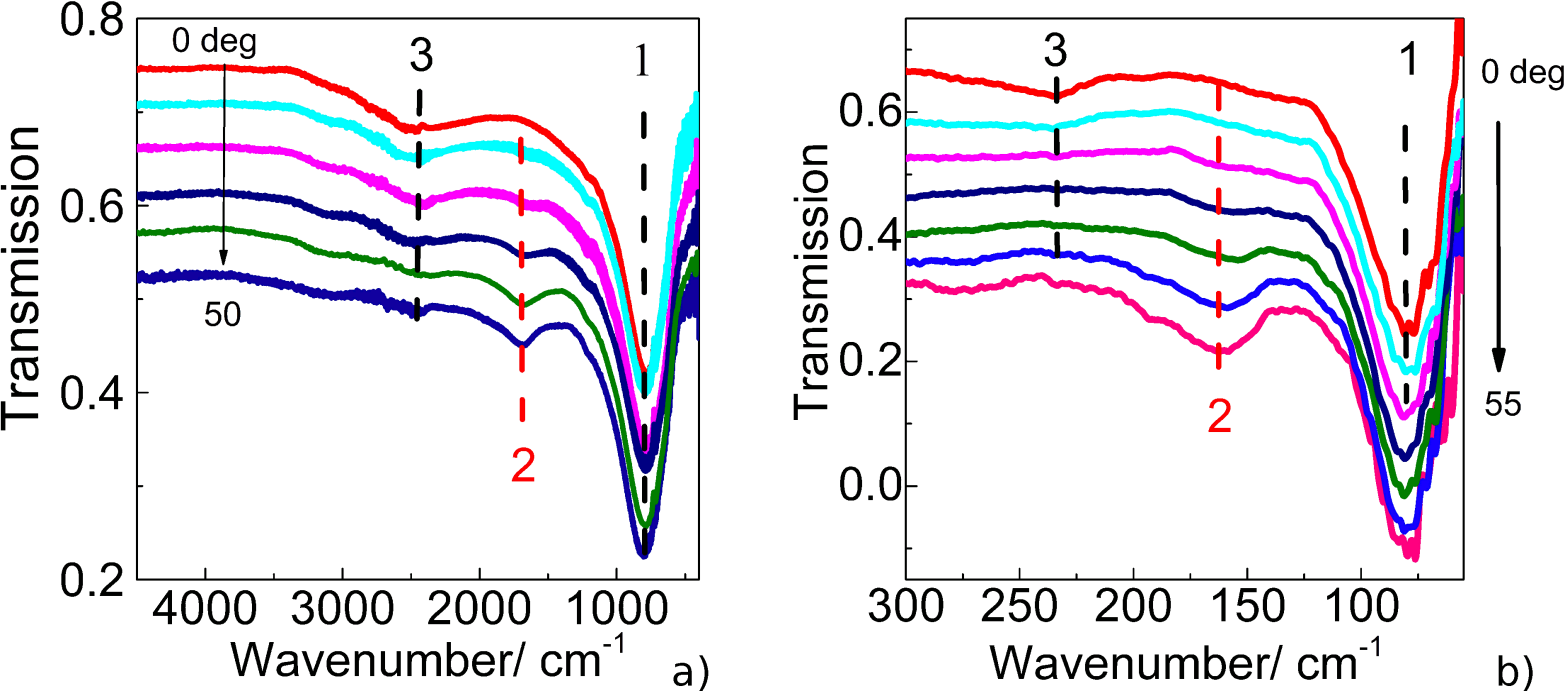
IR transmission spectra of the array of a) nanoantennas and b) microantennas measured at different angles of incidence, TM polarization. The vertical dashed lines with numbers indicate the positions of the LSPR modes of the corresponding order.

It should be noted that the even higher-order LSPR modes are not observed in the IR spectra of linear antennas under normal illumination due to the vector symmetry of the surface currents induced by the incident wave. Such an effect is known for microwave frequency selective surfaces [14] for which the LSPR mode can be interpreted as a standing wave excited on the structure's unit cell. For even modes, different parts of the standing wave tend to oscillate in antiphase such that the locally induced dipole moment of the cell is minimized and thereby prevents manifesting the LSPR features in the far field. Though the even LSPR modes are considered to be “dipole forbidden”, they can be activated by breaking the current’s symmetry. The simplest way to the symmetry breakdown is to deform the nanoantenna shape. Such a defect-induced activation of the 2nd-order LSPR mode was observed for non-ideal nanoantennas at normal incidence [37]. The second way to the symmetry breakdown condition is off-normal illumination [38]. In our work this effect was experimentally studied both for nano- and microantennas as shown in Figure 5a and 5b, respectively. These graphs demonstrate the angle-induced excitation of the 2nd-order LSPR modes, the intensity of which increases when the angle of incidence augments. In this case, the even LSPR modes appear due to a “retardation effect”, which implies that the incident wavefront reaches different points of the antenna at different times, thus inducing out-of-phase ohmic currents in the antenna metal and yielding the non-compensated dipole moment.

**Figure 6:**
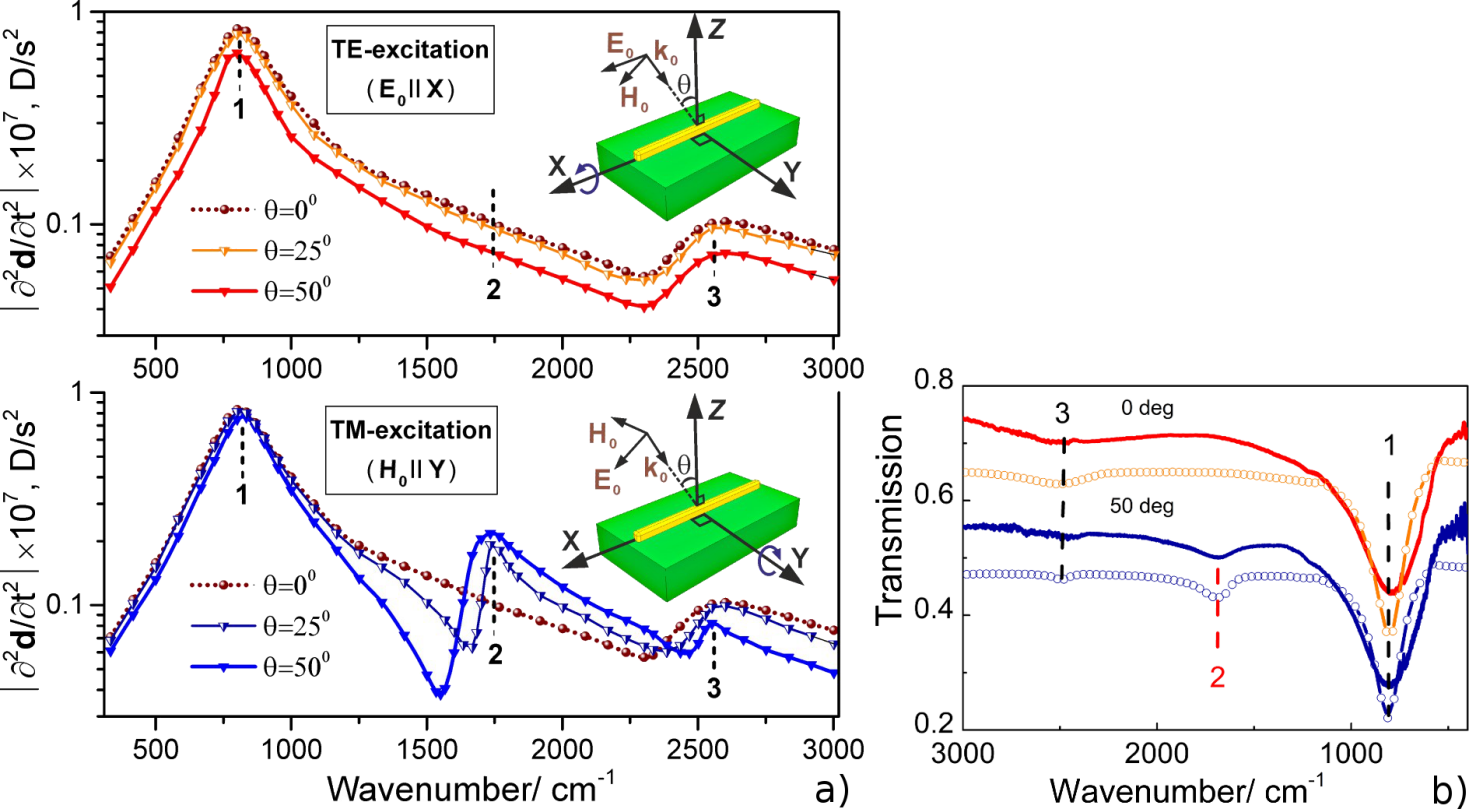
a) Evaluating the spectral behavior of the dipole radiation from a unit cell of the Si-backed array of linear nanoantennas at different angles of incidence θ and different polarizations: antenna length – 1800 nm, Y-period – 5000 nm. The graphs correspond to the wave power of *P*_0_ · cosθ with *P*_0_ = 1 W impinging upon the unit cell. b) Experimental (solid lines) and simulated (circles) SEIRA spectra of the linear nanoantennas for angles of incidence θ = 0 and 50° and TM polarization. The vertical dashed lines with numbers indicate the positions of the LSPR modes of the corresponding order.

To quantitatively illustrate the “retardation effect” in terms of the induced dipole moment under oblique illumination, in Figure 6a we present the results of simulating the magnitude of the second time derivative 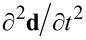 of the dipole moment **d** as a function of frequency by the example of linear nanoantennas with a length of 1800 nm. The results are obtained for three different angles of incidence θ = 0, 25, and 50°. Since the longitudinal LSPR mode can be excited either by TE- (**E_0_** II **X**) or TM-polarized (**H_0_** II **Y**) waves, both geometries are considered in the simulations. Note that switching to the second derivative of **d** instead of the dipole moment itself is explained by the fact that the far field radiation that is scattered (re-emitted) by the nanoantenna array is summed from the dipole radiation of individual antennas, where a specific intensity per unit solid angle is governed by the classical formula [39]:


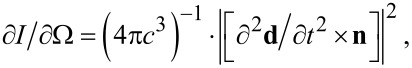


where *c* is the speed of light and **n** is the unit radius vector directed from the nanoantenna to the observation point. The simulations were accomplished with the help of ANSYS EM Suite R18 software, wherein the regime of Floquet ports and periodic boundary conditions was employed to model the nanoantenna array as a uniform periodic structure, while a Drude model with linear plasma and damping frequencies of 72,500 cm^−1^ and 216 cm^−1^, respectively, was applied to correctly describe the frequency response of gold [12]. To avoid undesirable computational effects arising from wave interference in the Si wafer of finite thickness, the Si medium supporting the nanoantenna array was assumed to fill semi-infinite space. This was implemented in ANSYS EM Suite by allowing the Si medium to touch one of the Floquet ports. In simulations, the procedure of adaptive meshing was accomplished at the highest frequency in the region of interest (3000 cm^−1^) that required taking into account 98 non-evanescent Floquet modes for the Si-touching Floquet port and 14 ones for the vacuum-touching port. When numerically computing the quantity of 
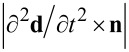
, its value was determined with the ANSYS built-in field calculator via integrating the complex vector magnitude of the ohmic current density **j**(**t**) over the nanoantenna volume according to the formula:


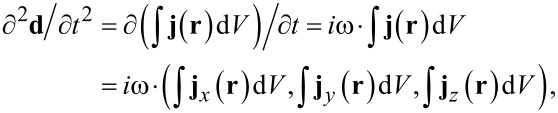


where ω is the radiation angular frequency, *i* is the imaginary unit.

The results presented in Figure 6 clearly show that LSPR modes are present in the dipole moment spectra as peaks of decaying amplitudes. For TE polarization, the electric field of which is parallel to the nanoantenna axis (**E_0_** II **X**) at any θ, there is no retardation effect and the even the LSPR mode remains dipole inactive. For TM polarization, the situation is fundamentally different: due to the appearance of the nonzero *z*-component of the electric field under oblique illumination, different parts of the antenna are excited at different times, thus inducing a non-compensated dipole moment for the even order LSPR mode and making it dipole active.

### SEIRA by semiconductor NCs on Au nanoantenna arrays

**Figure 7:**
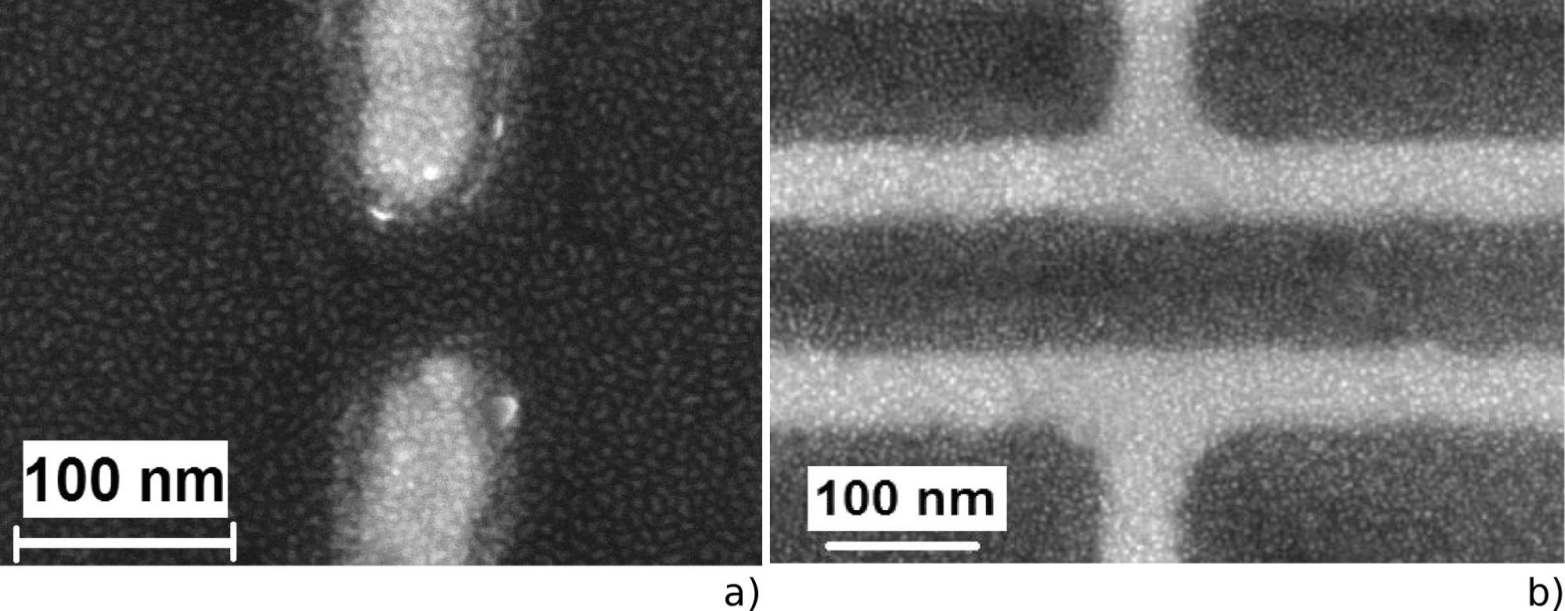
SEM images of the nanoantenna edges for a) linear and b) H-shaped nanoantennas taken after deposition of 1 ML of CdSe NCs. Figure 7a is reprinted with permission from [23], copyright 2017 American Chemical Society.

As it was shown in [21], the SEIRA spectra of 1 ML of CdSe NCs deposited on Au nanoantenna arrays reveal the fundamental surface optical (SO) mode of CdSe NCs at 190 cm^−1^. Hence, the optimal structural parameters of nano- and microantennas were determined from the calculations to ensure LSPR energies corresponding to SO phonons in CdSe (190 cm^−1^) [40–42], CdS (270 cm^−1^) [43], and PbS (190 cm^−1^) [43–44] NCs, and thereafter antenna arrays with the corresponding structural parameters, and consequently with required LSPR energies, were fabricated. Variants of the antenna arrays, including linear-shaped nano- and microantennas and H-shaped nanoantennas, were used for further SEIRA investigations. These arrays with appropriate structural parameters were also used for depositing CdSe, CdS, and PbS NCs in the Langmuir–Blodgett experiments. As it is seen from representative SEM images in Figure 7, a homogeneous monolayer of CdSe NCs is formed near the gaps of linear and H-shaped nanoantennas where the maximal ﬁeld enhancement for the light polarization along the nanoantennas is expected.

**Figure 8:**
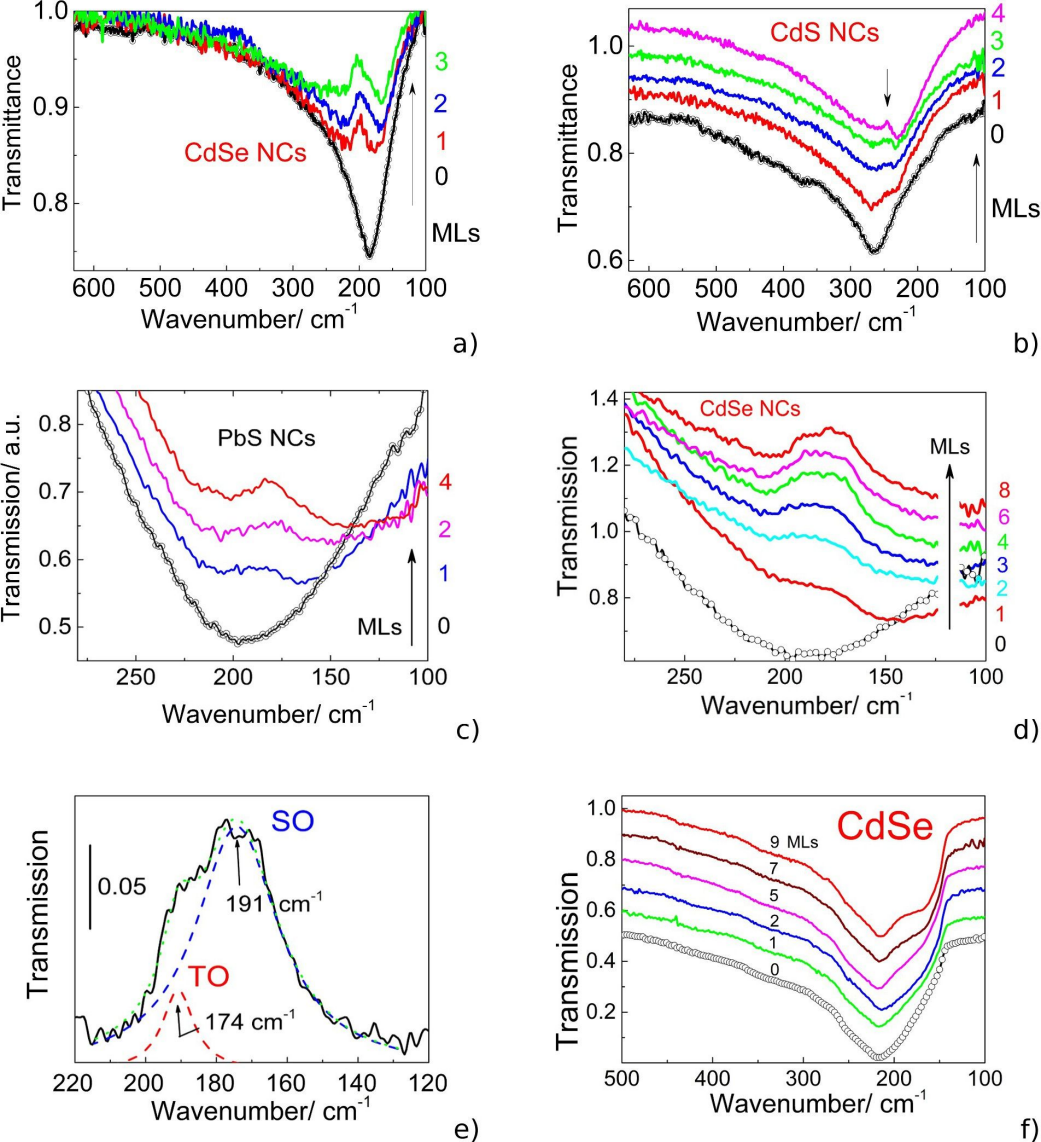
IR transmission spectra of the linear nanoantenna arrays before nanocrystal (NC) deposition (black circles) and after covering with different ML quantities for (a) CdSe and (b) CdS NCs. IR transmission spectra of H-shaped nanoantenna arrays before NC deposition (black circles) and after covering with different ML quantities for (c) PbS and (d) CdSe NCs. e) Fragment of the IR spectrum of 9 MLs of CdSe NCs deposited on the H-shaped nanoantenna arrays (Figure 8d, curve 8) after background subtraction. f) IR transmission spectra of microantenna arrays before NC deposition (black circles) and after covering with different ML quantities for CdSe NCs. Figure 8a and 8b are reprinted with permission from [23], copyright 2017 American Chemical Society.

The IR transmission spectra of the samples with linear nanoantenna arrays before and after deposition of CdSe and CdS NCs with different ML quantities were recorded and are presented in Figure 8a and Figure 8b, respectively. The IR spectra of the as-prepared nanoantennas reveal sharp minima near 190 or 265 cm^−1^ in accordance with the LSPR energies predicted by the simulations.

The deposition of 1 ML of CdSe and CdS NCs on linear nanoantennas (Figure 8a and 8b) as well as of PbS and CdSe NCs on the H-shaped nanoantennas (Figure 8c and 8d) induces clearly resolved features in the SEIRA spectra near 190 and 250 cm^−1^, respectively, which are superimposed onto the LSPR minima attributed to the SO modes of the NCs. With increasing number of deposited NC monolayers, their intensity increase entails a high frequency shift of the LSPR minima, which is more pronounced for H-shaped nanoantennas (Figure 8c and 8d). This shift occurs due to the change in dielectric function of the medium surrounding the nanoantennas. At a relatively thick CdSe NC coating on H-shaped nanoantenna arrays, the SEIRA response by NCs consists of at least two obvious components of the SO mode: one at 191 cm^−1^ and a weaker feature at 171 cm^−1^. Their frequencies were determined from the best fit using two Lorentzian curves. The appearance of the latter feature is attributed to the absorption by TO phonon modes in CdSe NCs normally active in IR spectra. As it was shown in our earlier paper [23], the intensity of the SEIRA response of NCs on a linear nanoantenna array can be maximized by a proper choice of the array period when the energy of a diffraction mode coincides with that of the LSPR mode. Note, however, that the overall intensities of the phonon modes of NCs deposited on the linear and H-shaped nanoantenna arrays are comparable. Thus, the local SEIRA enhancement of linear nanoantennas appears to be significantly larger than that of H-shaped nanoantennas due to the lower values of the electromagnetic field localized between the cross-arms of H-shaped elements. The comparable intensities of the phonon modes of NCs deposited on the linear and H-shaped nanoantenna seen in Figure 8a and 8d, respectively, are not consistent with the calculations predicting the higher gain (175%) for the H-shaped nanoantennas relative to that of the linear ones. The reason for such a discrepancy between the theoretical expectations and experimental data is most likely explained by lower areal density of the NC located in the gap between the cross-arms of the H-shaped antennas that originates from the LB deposition process.

## Conclusion

We report on systematic experimental and theoretical investigations of mid- and far-infrared LSPRs in arrays of linear and H-shaped Au nanoantennas and linear Au microantennas. We demonstrate comparative SEIRA experiments with semiconductor NCs deposited on the antenna arrays with the optimal structural parameters. This ensures that the LSPR frequencies are adjusted to the range of optical phonons in semiconductor NCs. We show that although the overall IR response by NCs deposited on Au antenna arrays with different morphologies is comparable, the maximal SEIRA enhancement is achieved for the linear geometry due to a highly localized electromagnetic field between the nanoantenna edges.
